# Intestinal Obstruction

**Published:** 1914-06

**Authors:** 


**Intestinal Obstruction.** David M. Davis and Harold S. Morgan, Bulletin of the Johns Hopkins Hospital, February, 1914; review the literature and detail various experiments. The articles are not well adapted to abstracting but the authors conclude that the gravity of the condition is due largely to toxins and that these are not, as might be imagined, of bacterial origin, but rather due to some perverted action of the cells of the mucosa.

